# CHILDHOOD PARENT–CHILD RELATIONSHIP, PARTNER RELATIONSHIP CLOSENESS, AND DEPRESSIVE SYMPTOMS IN LATER LIFE

**DOI:** 10.1093/geroni/igad104.3033

**Published:** 2023-12-21

**Authors:** Zexi Zhou, Yang Qu

**Affiliations:** The University of Texas at Austin, Austin, Texas, United States; Northwestern University, Chicago, Illinois, United States

## Abstract

Attachment theory suggests that quality of early-life parent-child relationships may influence how people establish other types of relationships (e.g., romantic relationship, friendship) later in life, and have long-lasting effects on individuals’ psychosocial development. Yet, little research has investigated whether such effects may last until later adulthood and the potential moderators in these processes. To address this issue, this study examined the associations among childhood parent-child relationship quality, current partner relationship closeness, and depressive symptoms in late life using a sample of 12,292 community-dwelling adults aged 51 or older (Mage = 63.15, 51% female). We leveraged the seven-wave longitudinal data between 2006–2018 from the Health and Retirement Study. Depressive symptoms were assessed using the CESD score at each wave. Participants also reported closeness with their partner every other wave, and relationship quality with their parents in childhood. Multilevel models showed that better mother-child and father-child relationships during childhood were both associated with greater closeness with partner in late life, especially for female compared to male. Poorer childhood mother-child relationship was associated more depressive symptoms in late life, but this association was attenuated for those who overall reported a greater level of closeness with their partner over the study period. The findings suggest that negative early life parent-child relationship may put people at greater risk for developing depressive symptoms and weaker social connections in late life, and highlight the protective role of positive partner relationship in buffering this association.

